# Emodin alleviates lung ischemia–reperfusion injury by suppressing gasdermin D‐mediated pyroptosis in rats

**DOI:** 10.1111/crj.13582

**Published:** 2023-02-07

**Authors:** Tao Jin, Fen Ai, Jin Zhou, Lin Kong, Zhangming Xiong, Dingping Wang, Ruilin Lu, Zhen Chen, Muxi Zhang

**Affiliations:** ^1^ Department of Anesthesiology Suining First People's Hospital Suining Sichuan China; ^2^ Department of Emergency, The Central Hospital of Wuhan, Tongji Medical College Huazhong University of Science and Technology Wuhan China; ^3^ Department of Proctology Suining First People's Hospital Suining Sichuan China; ^4^ Department of Ophthalmology Suining First People's Hospital Suining Sichuan China

**Keywords:** emodin, gasdermin D, lung ischemia–reperfusion, NLRP3, pyroptosis

## Abstract

**Background:**

Pyroptosis refers to programmed cell death associated with inflammation. Emodin has been reported to alleviate lung injuries caused by various pathological processes and attenuate ischemia–reperfusion (I/R) injuries in diverse tissues.

**Methods:**

Lewis rats were assigned into the sham, the I/R, and the I/R + emodin groups. Emodin and phosphate‐buffered saline were intraperitoneally injected into rats of the emodin group and I/R group for 30 min, respectively. These rats were then subjected to left thoracotomy followed by 90‐min clamping of the left hilum and 120‐min reperfusion. Sham‐operated rats underwent 210‐min ventilation. Lung functions, histological changes, lung edema, and cytokine levels were assessed. Protein levels were measured by western blotting. Immunofluorescence staining was conducted to evaluate pyroptosis.

**Results:**

Emodin alleviated the I/R‐induced lung dysfunction, lung damages, and inflammation. Protective effects of emodin against I/R‐mediated endothelial pyroptosis was observed in vivo and in vitro. Mechanistically, emodin inactivated the TLR4/MyD88/NF‐κB/NLRP3 pathway.

**Conclusion:**

Emodin attenuates lung ischemia–reperfusion injury by inhibiting GSDMD‐mediated pyroptosis in rats.

## Graphical abstract

During ischemia and reperfusion, pyroptosis, which exhibits morphological characteristics of apopotsis and inflammation, plays an important role in lung injury. Emodin is found to inhibit pyroptosis via inactivation of NLRP3/caspase‐1/GSDMD and TLR4/MyD88/NF‐kB pathways in cellular and animal models of lung ishcemia‐reperfusion injury.

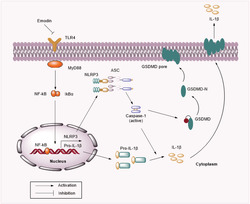

## 1 INTRODUCTION

Ischemia–reperfusion injury occurs when the blood supply is temporarily interrupted and leads to organ damage. Lung ischemia–reperfusion injury (LIRI) is a life‐threatening surgical emergency that occurs in diverse clinical situations, including cardiopulmonary bypass cardiac surgery,^1^ lung transplantation,^2^ high‐volume resuscitation,^3^ pulmonary embolism,^4^ and single‐lung ventilation.^5^ The development of LIRI results in prolonged hospital day, increased medical expenditures, and high morbidity and mortality.^6^ Currently, effective methods to LIRI treatment remain inadequate. Thus, investigation of novel therapeutic options to prevent LIRI is necessary.

Pyroptosis refers to programmed cell death associated with inflammation.^7^ The features of cell lysis, cell swelling, and inflammatory response attribute to its distinction from apoptosis and necroptosis.^8^ The canonical pyroptosis pathway is mediated by caspase‐1 and cleaved gasdermin D (GSDMD).^9^ The NLRP3 inflammasome complex can promote pro‐caspase‐1 activation through self‐cleavage into active caspase‐1 and then cleaves GSDM family proteins, leading to pyroptosis.^10^ NLRP3, pro‐caspase‐1, and apoptosis‐associated speck‐like adaptor protein (ASC) are major components of the NLRP3 inflammasome.^11^ Activated caspase‐1 cleaves the precursors of IL‐1β and IL‐18 into mature IL‐1β and IL‐18, which activate the immune response and induce inflammation.^12^ GSDMD is cleaved by inflammatory caspases between its N‐terminal gasdermin‐N (GSDMD‐N) and C‐terminal gasdermin‐C domains.^13^ The GSDMD‐N moving to the plasma membrane forms a pore, causing the leakage of IL‐1β.^9^ LIRI could be attenuated by suppressing selective NLRP3 inflammasome and pyroptosis.^14^, ^15^ Thus, inhibition of NLRP3 inflammasome and pyroptosis is beneficial to alleviate LIRI.

Emodin derived from *Rheum palmatum* is commonly used as a laxative in traditional Chinese medicine.^16^ As reported, emodin could alleviate lung injuries caused by various pathological activities. For example, emodin ameliorates lung injury caused by intestinal damage via the TLR4/NF‐κB and SP‐A pathways.^17^ Emodin‐induced inhibition of NLRP3 inflammasome alleviates the pancreatitis‐associated lung injury.^18^ In animals with lipopolysaccharide (LPS)‐mediated lung injury, emodin ameliorates pulmonary inflammation via the mTOR/HIF‐1α/VEGF pathway.^19^ Emodin can attenuate lung injury by promoting autophagy in mice with endotoxemia.^20^ Additionally, accumulating evidence have suggested the protective roles of emodin in ischemia–reperfusion (I/R) injuries. For example, emodin attenuates renal I/R injury by inhibiting mitochondrial fission.^21^ Pretreatment of emodin ameliorates the myocardial I/R injury.^22^ Moreover, emodin administration inhibits neuronal apoptosis and alleviates oxidative stress through the ERK1/2 signaling in rats with cerebral I/R.^23^ However, the functions of emodin and its related mechanisms underlying LIRI remain uncertain.

TLR4/MyD88/NF‐κB are critical players in activating NLRP3 inflammasome.^24^ As pattern‐recognition receptors, Toll‐like receptors (TLRs) serve as defenders against endogenous danger and microbial infection. Danger‐ and pathogen‐associated molecular patterns (PAMPs/DAMPs) are involved in this defending process by interacting with TLRs.^25^ PAMP recognition by TLRs causes the recruitment of intracellular Toll/interleukin‐1 receptor (TIR) domain‐containing adaptor proteins among which myeloid differentiation primary response gene 88 (MyD88) is the first to be identified.^26^ The recruitment of MyD88 promotes downstream activation of NF‐κB.^27^ Emodin prevents intestinal and lung damages via the TLR4/NF‐κB pathway,^17^ and alleviates LPS‐stimulated myocardial injury by inactivating the NLRP3 inflammasome.^28^ Thus, this study investigates the biological functions of emodin and detects whether the functions are mediated by the TLR4/MyD88/NF‐κB/NLRP3.

## 2 METHODS

### 2.1 Animals

Lewis rats (male, 11 weeks of age, 260–290 g; Charles River Laboratories, Beijing, China) were housed at 25 ± 2 °C with 50%–60% humidity under a cycle of 12‐h light/12‐h dark. All rats were available to food and water. Experimental protocols were granted approval from the Ethics Committee of Wuhan Myhalic Biotechnology Co., Ltd (approval number: 202104099).

### 2.2 Experimental protocols

**FIGURE 1:**
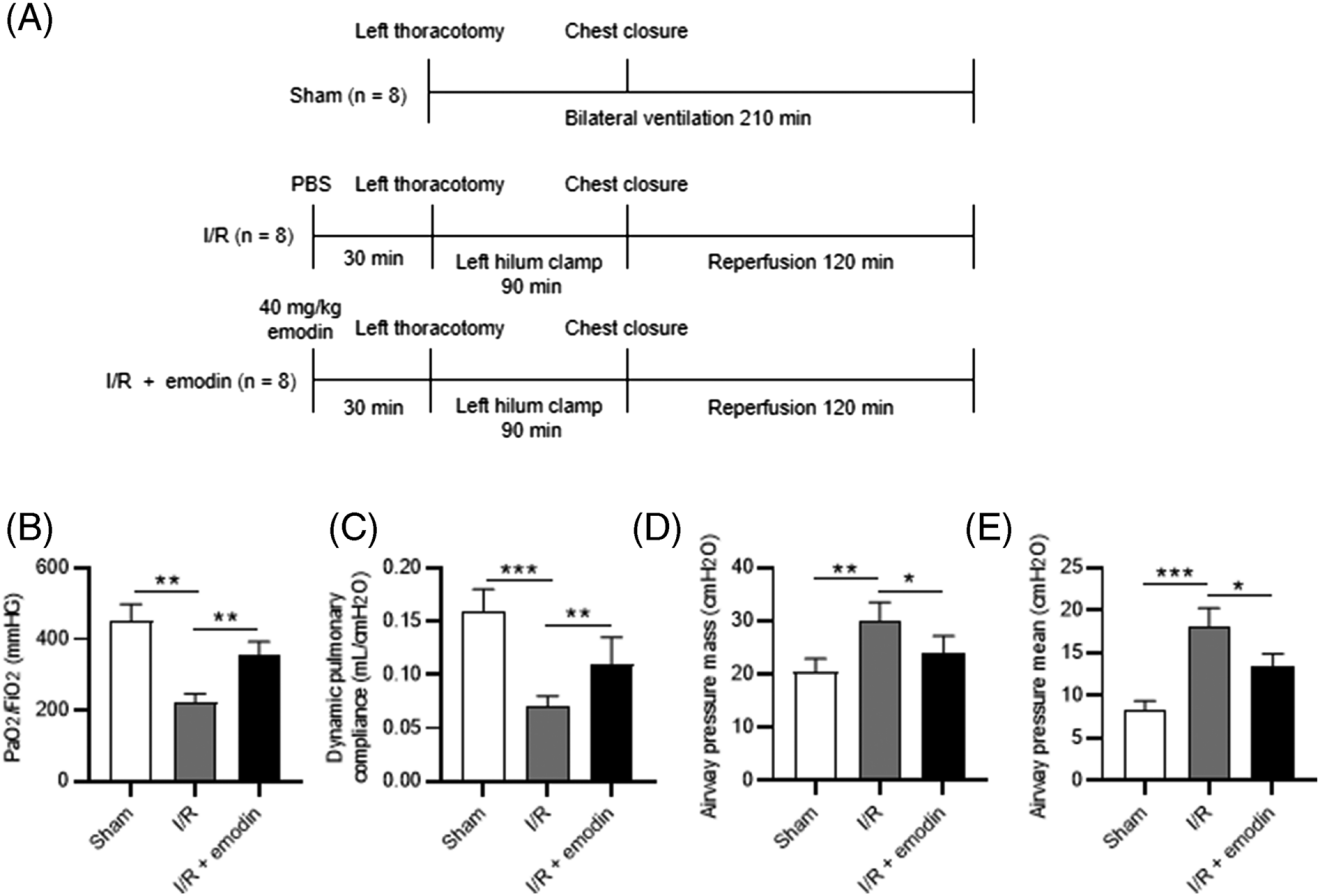
Emodin alleviates the I/R‐induced lung dysfunction. (A) Diagram of the experimental protocols. (B) Arterial partial pressure of oxygen/fractional inspired oxygen (PaO_2_/FiO_2_). (C) Dynamic pulmonary compliance. (D) Peak airway pressure. (E) Mean airway pressure. The results are expressed as the mean ± standard deviation (*n* = 8 rats per group). All experiments were performed in triplicate. *p < 0.05, **p < 0.01, ***p < 0.001

The rats were assigned into the sham, the I/R, and the I/R + emodin groups (*n* = 8). Experimental procedures were briefly presented in Figure 1A. Before the surgery, animals were anaesthetized by intraperitoneal injection of sodium pentobarbital (100 mg/kg). Heparin (1000 U/kg) was initially injected via the jugular vein. Sham‐operated rats only underwent thoracotomy (left lung) and 210‐min ventilation. Rats in the I/R + emodin group were injected intraperitoneally with emodin (40 mg/kg) (MedChemExpress, Shanghai, China), while those in I/R group were injected intraperitoneally with phosphate‐buffered saline (PBS; BioSun, Shanghai, China). After 30‐minitue administration of emodin or PBS, left thoracotomy was performed. The dosage of emodin was determined following a previous study.^19^ Subsequently, the left hilum was occluded using a small metallic clamp for 90 min. The tidal volume (TV) was decreased to 6 ml/kg until reperfusion. The clamp was released following 90 min of occlusion, and reperfusion was conducted through restoration of blood flow and ventilation to the bilateral lung for 120 min. Ventilation was started using a Model SN‐480‐7 (Shinano Seisakujo, Tokyo, Japan). During the process of bilateral lung ventilation, the TV and the respiratory rate were set to 9 ml/kg and 60 breaths per minute, respectively. Throughout the experiment, the fraction of inspired oxygen was kept at 100%, and positive end‐expiratory pressure was maintained at 2‐cm H_2_O.

### 2.3 Lung mechanics measurements

After 120 min of reperfusion, lung injury was assessed by occluding the right hilum through a median sternotomy. The TV was reduced to 5 ml/kg and the respiratory rate was 60 breaths per minute. For arterial blood gas analysis, blood samples were collected after 5 min of ventilation through the ascending aorta. The rats were subsequently connected to the flexiVent system (SCIREQ, Montreal, Canada). Physiologic data, dynamic compliance, and airway pressures was measured by alternating perturbations of the single forced oscillation families in a closely spaced manner (Snap‐Shot, SCIREQ).

### 2.4 Lung edema

Tissue samples were obtained from the left lung after measurement of pulmonary function and then divided into three parts. The estimation of wet‐to‐dry ratio was performed using the upper section. Evaluation of wet weight was soon after the harvest. After desiccating at 100 °C for 24 h, the dry weight was obtained. The formula was as follows: wet‐to‐dry ratio = wet weight/dry weight.

### 2.5 Hematoxylin–eosin (H&E) staining

The middle part of tissue samples was fixed in 4% paraformaldehyde, embedded in paraffin, and cut into sections with 1‐μm thickness. Then, the sections were stained with H&E (Yuanye Biotechnology, Shanghai, China) to examine histological changes and imaged using a light microscope (Olympus, Tokyo, Japan). Two blinded pathologists participated in the assessment of the degree of lung injury. A 4‐point scale was used: none (0), mild (1), moderate (2), or severe (3). The lung injury score was obtained by summing the resulting two scores.^29^

### 2.6 Cell culture and treatment

Pulmonary microvascular endothelial cells (PMVECs; Sunncell, Wuhan, China) were cultured in 89% DMEM (Sunncell) supplemented with 10% FBS (Sigma‐Aldrich), 0.5% penicillin and 0.5% streptomycin in a humidified atmosphere with 5% CO_2_ at 37 °C.

Cells were grouped into the control, the oxygen/glucose deprivation and reoxygenation (OGD/R), the OGD/R + emodin and the OGD/R + Bay‐117082. The PMVECs were subjected to PBS washing for three times, fed medium without glucose and serum, and then placed for 1 h at 37 °C in a Whitley H35 Hypoxystation (Hua Yue, Guangzhou, China) in an atmosphere of 1% O_2_, 5% CO_2_, and 94% N_2_. Subsequently, the cells were cultured for 4 h at 37 °C in glucose‐containing medium in an atmosphere of 5% CO_2_ and 95% O_2_.^30^ The cells in the OGD/R + emodin group were pretreated with 40‐μM emodin 1 h before reoxygenation. In the OGD/R + Bay‐117082 group, the cells were pretreated with 5‐μM Bay‐117082 (Selleck Chemicals, Shanghai, China) for 1 h before reoxygenation.

### 2.7 Enzyme‐linked immunosorbent assay (ELISA)

The lower part of the left lung (10 mg) was homogenized with 500 μl of PBS with protease inhibitors (Selleck Chemicals), followed by being centrifuged at 10000*g* for 20 min at 4 °C. The concentrations of TNF‐α, IL‐1β, and IL‐6 in tissue lysates, rat serum, or cell supernatant were measured using ELISA kits (Bio‐Techne, Shanghai, China) according to the manufacturer's instructions.

### 2.8 Western blotting

Tissue and cellular samples were homogenized in RIPA lysis buffer (Beyotime, Shanghai, China) with phosphatase inhibitor cocktail and protease inhibitors (MedChemExpress). Protein concentration of extracts of PMVECs or lung tissue homogenates was examine using a BCA protein assay kit (Beyotime). Protein samples (15 μg) were loaded on sodium dodecyl sulfate‐polyacrylamide gel electrophoresis (SDS‐PAGE) and transferred onto a polyvinylidene fluoride (PVDF) membrane. After blocking with 5% skimmed milk for 1 h, the membrane was incubated overnight with primary antibodies against ASC (ab180799, 1:1000; Abcam), caspase‐1 p20 (WL02996a, 1:1000; Wanleibio, Shenyang, China), MyD88 (ab219413, 1:1000; Abcam), procaspase‐1 (ab179515, 1:1000; Abcam), GSDMD‐N (#397545, 1:1000; Cell signaling Technology, Shanghai, China), NLRP3 (ab263899, 1:1000; Abcam), pro‐GSDMD (#464515, 1:1000; Cell signaling), GAPDH (ab181602, 1:10000; Abcam), TLR4 (sc‐293 072, 1:1000; San Cruz Biotechnology, Dallas, TX, USA), p‐p65 (ab76302, 1:1000; Abcam), and p65 (ab19870, 2.5 μg/ml; Abcam) at 4 °C. The membrane was then washed and incubated with secondary antibodies (1:20000) for 1 h at room temperature. An enhanced chemiluminescence detection system (Yeasen, Shanghai, China) was used to detect antibody‐specific proteins. Band densitometry analysis was performed using ImageJ software.

### 2.9 Immunofluorescence staining

PMVECs were fixed with 4% paraformaldehyde for 1 h followed by three‐time PBS washing. The samples were then permeabilized with 0.1% Triton X‐100 (Sigma‐Aldrich) for 20 min and blocked with 5% bovine serum albumin (Sigma‐Aldrich) for 60 min. Subsequently, the samples were incubated overnight with primary antibody against GSDMD (A18281, 1:100; ABclonal, Wuhan, China) at 4 °C, followed by being incubated with secondary antibody (Alexa Fluor 488; Beyotime) for 1 h at room temperature in the dark. DAPI (ab228549; Abcam) was used for nuclear staining. The staining pattern was imaged using a fluorescence microscope (Olympus).

### 2.10 LDH release rate

To measure LDH release in the culture supernatant, a lactic dehydrogenase (LDH) working solution (ChemeGen, Shanghai, China) was used. The cells were adjusted to 1 × 10^4^ cells/ml and then inoculated in a 96‐well plate I (100 μl/well). Subsequently, 100‐μl LDH working solution was added and the plate was gently shaken at room temperature for 40 min. Then the medium was seeded in a new 96‐well culture plate II, and 10‐μl LDH reaction solution was added into the new plate. The LDH release rate was calculated by the formal: LDH release (%) = optical density (OD) II/ (OD II − OD 1) × 100%. The experiment was done in triplicate.

### 2.11 Statistics analysis

All experiments were performed at least three independent repeats. GraphPad Prism (GraphPad Software, San Diego, CA, USA) was used to analyze statistics. The data were described as the mean ± standard deviation. One‐way analysis of variance followed by Tukey's post hoc analysis and Student's *t* test were performed to compare differences. *p* < 0.05 was considered statistically significant.

## 3 RESULTS

### 3.1 Emodin alleviates the I/R‐induced pulmonary dysfunction

The experimental protocol is shown in Figure 1A. The I/R group exhibited lower arterial oxygenation and dynamic compliance than the sham group, which were enhanced by emodin administration (Figure 1B,C). Additionally, peak and mean airway pressures were exacerbated following I/R insult, while emodin treatment abolished the I/R‐induced promotion in the peak and mean airway pressures (Figure 1D,E). These results demonstrate that emodin administration improved the lung function of I/R‐treated rats.

### 3.2 Emodin ameliorates the I/R‐induced inflammation

**FIGURE 2:**
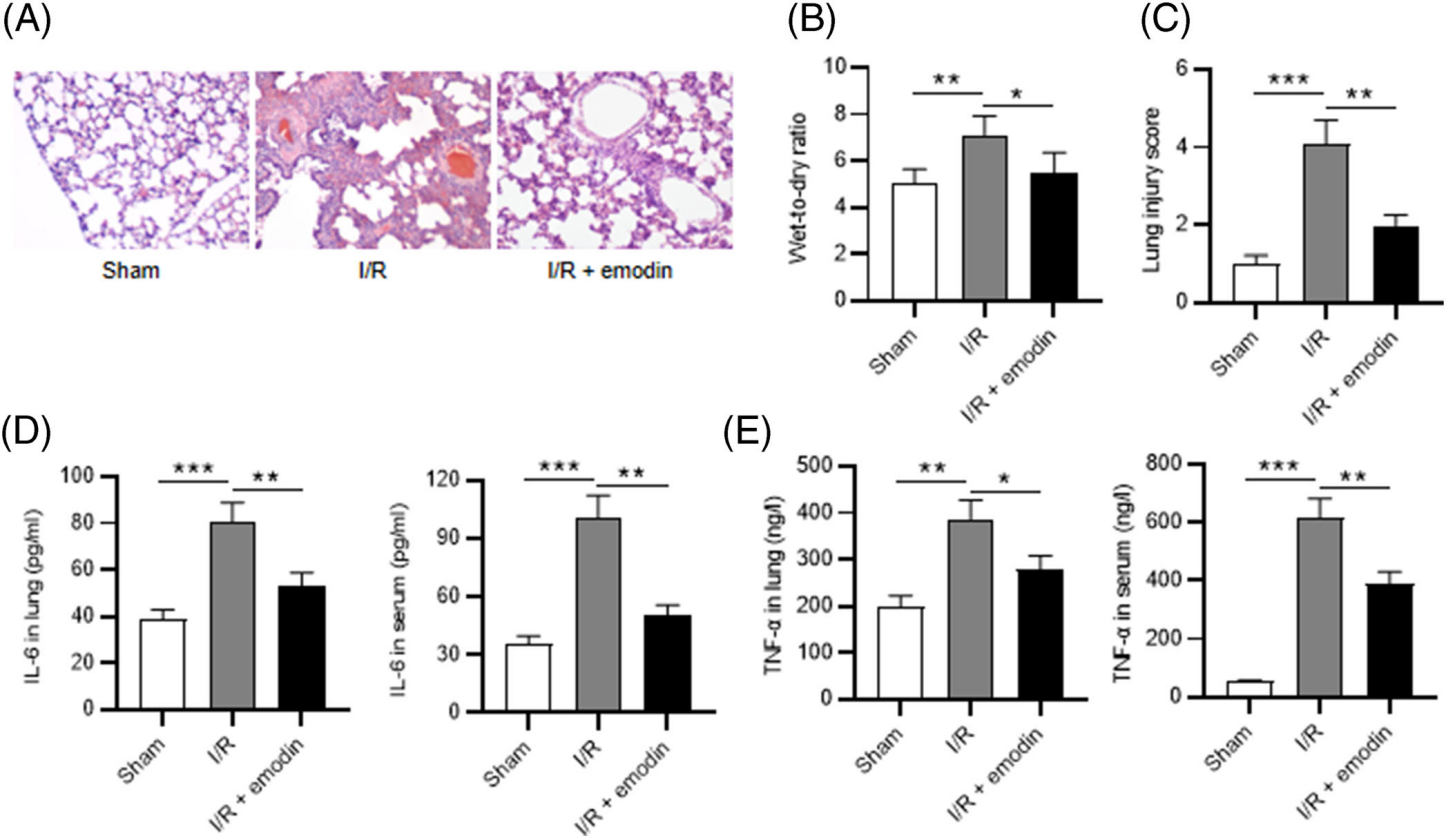
Emodin ameliorates the I/R‐induced lung injury and inflammation. (A) Representative lung tissue sections stained by H&E. (B) Wet‐to‐dry ratios in the sham, the I/R, and the I/R + emodin groups. (C) Lung injury scores. (D) Levels of IL‐6 and TNF‐α in lung tissues and serum. The results are expressed as the mean ± standard deviation (*n* = 8 rats per group). All experiments were performed in triplicate. *p < 0.05, **p < 0.01, ***p < 0.001

Lung tissue obtained from sham‐operated rats exhibited normal histologic results. However, intra‐alveolar hemorrhage, severe interstitial edema, and massive neutrophil infiltration were observed post‐I/R, which were ameliorated by emodin, as H&E staining revealed (Figure 2A). Additionally, perivascular edema was evaluated by the wet‐to‐dry weight ratio. The results showed that wet‐to‐dry ratio was markedly higher in the I/R group, while administration of emodin led to a decrease in the ratio, suggesting that emodin attenuated lung edema caused by I/R (Figure 2B). Simultaneously, emodin abolished the enhancing effect of I/R induction on lung injury scores (Figure 2C). Moreover, ELISA results showed that levels of IL‐6 and TNF‐α in lung tissues and serum were higher in the I/R group than in the sham group. However, emodin administration significantly decreased their levels (Figure 2D,E). Collectively, emodin alleviates pulmonary injury and inflammation in rats with I/R.

### 3.3 Emodin attenuates the I/R‐induced endothelial pyroptosis

**FIGURE 3:**
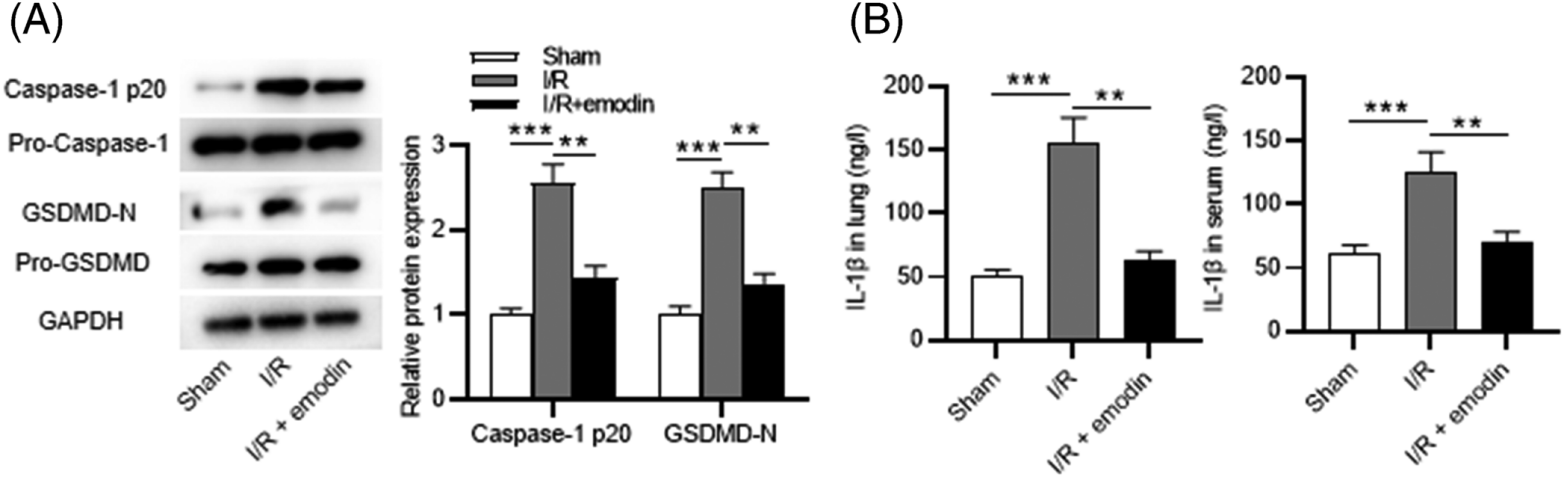
Emodin inhibits the I/R‐induced pyroptosis. (A) The protein levels of caspase‐1 p20 and GSDMD‐N in lung tissues detected by western blotting. (B) The level of IL‐1β detected by ELISA. The results are expressed as the mean ± standard deviation (*n* = 8 rats per group). All experiments were performed in triplicate. **p < 0.01, ***p < 0.001

Western blotting was conducted to measure the levels of pyroptosis markers in lung tissues to determine the protective effects of emodin against endothelial pyroptosis. The results demonstrated that I/R insult significantly elevated protein levels of caspase‐1 p20 and GSDMD‐N, which were decreased by emodin (Figure 3A). Additionally, the levels of IL‐1β in tissues and rat serum were increased post‐I/R, while emodin treatment counteracted the promoting effects of I/R insult (Figure 3B). These results indicate that emodin alleviates pyroptosis in I/R‐treated rats.

### 3.4 The pyroptosis of PMVECs is attenuated by emodin

**FIGURE 4:**
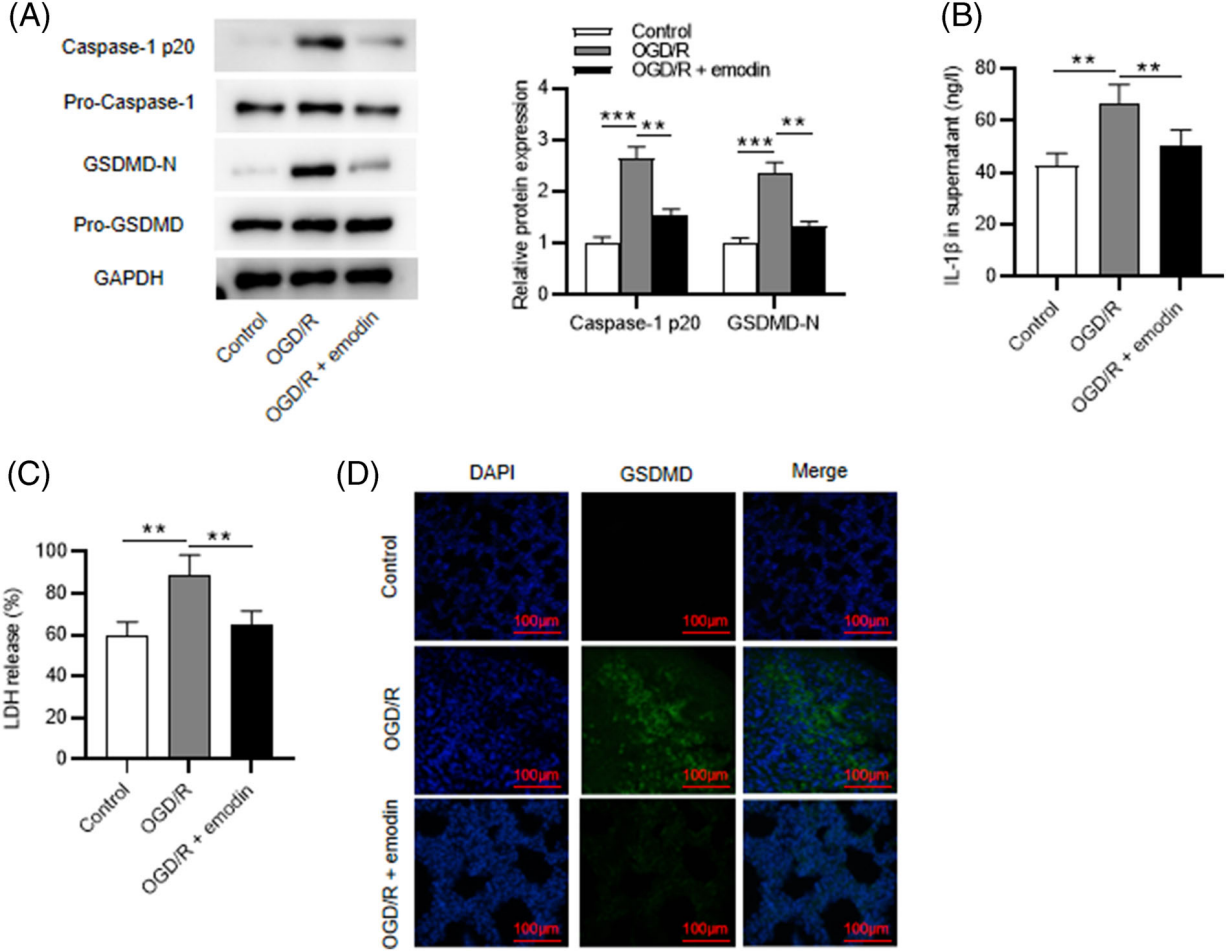
The pyroptosis of PMVECs is attenuated by emodin. (A) The protein levels of caspase‐1 p20 and GSDMD in PMVECs detected by western blotting. (B) Levels of IL‐1β in cell supernatant. (C) LDH release rate. (D) Immunofluorescence staining of GSDMD in PMVECs. DAPI was used to stain the cell nuclei. Scale bar, 200 μm. The results are expressed as the mean ± standard deviation. All experiments were performed in triplicate. **p < 0.01, ***p < 0.001

As western blotting revealed, the OGD/R‐induced promotion in protein levels of caspase‐1 p20 and GSDMD‐N was attenuated by emodin (Figure 4A). Additionally, IL‐1β expression was upregulated post‐OGD/R in cell supernatant, while emodin decreased its level, as ELISA showed (Figure 4B). As LDH release assays revealed, emodin restored the OGD/R‐induced elevation in LDH release (Figure 4C). Moreover, the results of immunofluorescence staining revealed that emodin treatment reversed the OGD/R‐induced upregulation in GSDMD expression (Figure 4D). Collectively, emodin alleviates OGD/R‐induced pyroptosis of PMVECs.

### 3.5 Emodin inactivates the TLR4/MyD88/NF‐κB/NLRP3 pathway

**FIGURE 5:**
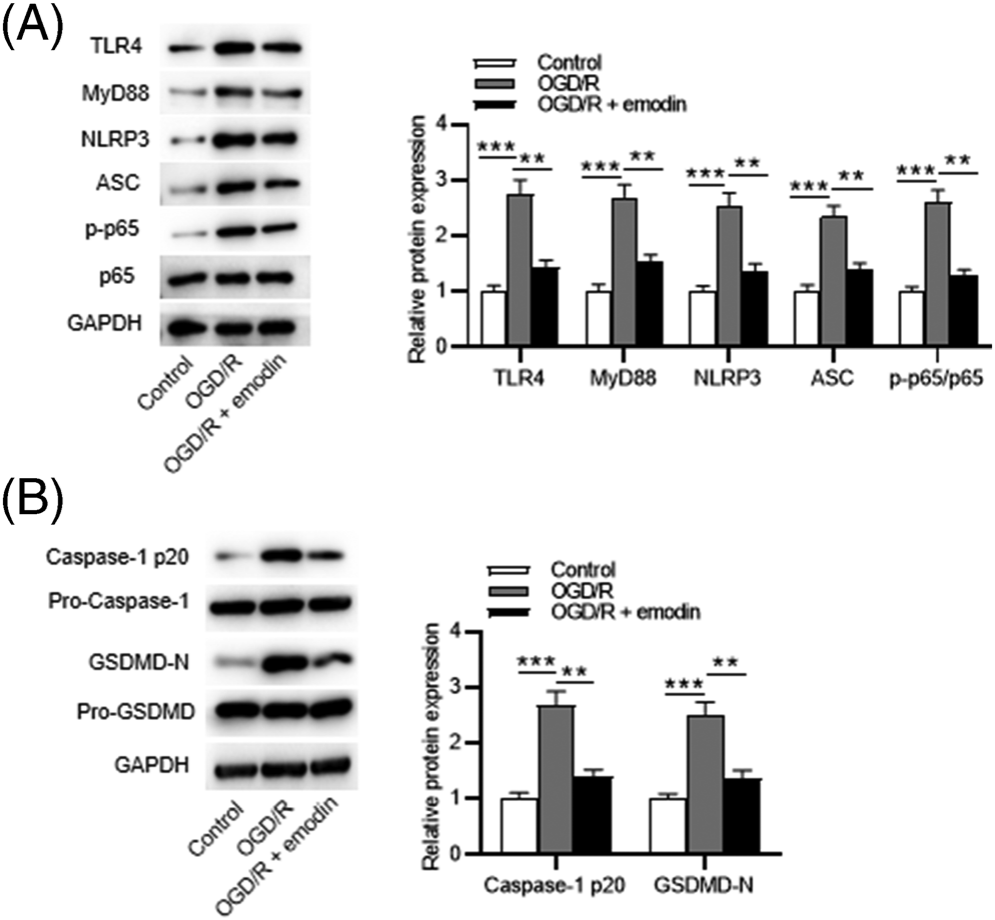
Emodin inactivates the TLR4/MyD88/NF‐κB/NLRP3 pathway. (A) Protein levels of TLR4, MyD88, ASC, p‐p65, and p65 in the control, the OGD/R, and the OGD/R + emodin groups. (B) Protein levels of caspase‐1 p20 and GSDMD‐N in the control, the OGD/R, and the OGD/R + Bay‐117082 groups. The results are expressed as the mean ± standard deviation. All experiments were performed in triplicate. **p < 0.01, ***p < 0.001

**FIGURE 6:**
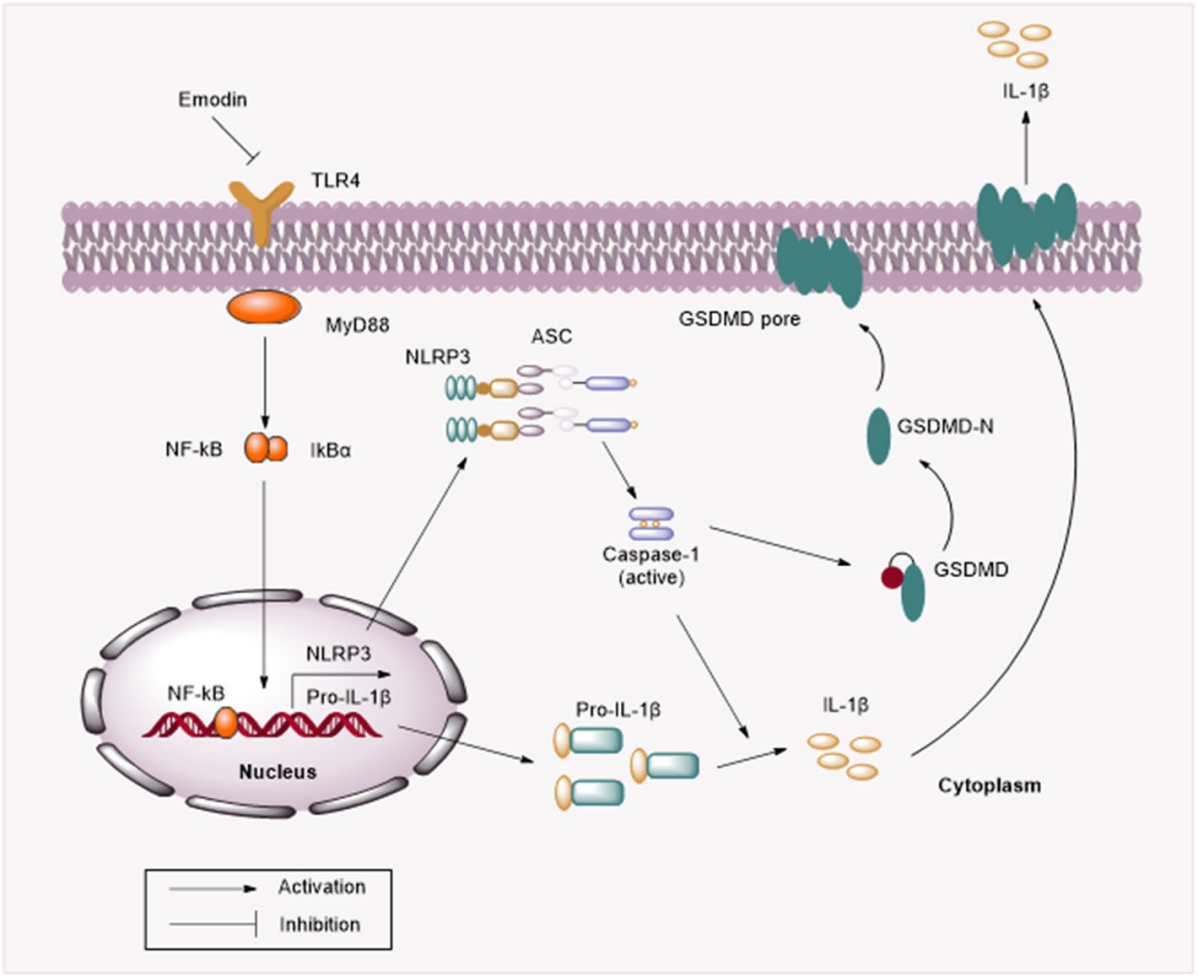
Schematic diagram depicting the underlying mechanisms by which emodin alleviates I/R‐induced pulmonary dysfunction

As western blotting showed, protein levels of TLR4, MyD88, NLRP3, and ASC and ratios of phosphorylated‐p65/p65 were significantly increased following OGD/R insult. However, emodin administration reversed the promoting effects of OGD/R (Figure 5A). Then we used Bay‐117082 (a NF‐κB pathway inhibitor) to detect whether NF‐κB inhibition could attenuate pyroptosis. The results showed that NF‐κB inhibition decreased protein levels of caspase‐1 p20 and GSDMD‐N, indicating that NF‐κB inhibition could inhibit the OGD/R‐induced pyroptosis of PMVECs (Figure 5B). Figure 6 shows the schematic diagram depicting the underlying mechanisms by which emodin alleviates I/R‐induced pulmonary dysfunction. In conclusion, this study demonstrates that emodin alleviates I/R‐induced pulmonary dysfunction by attenuating inflammation and pyroptosis through inactivation of the TLR4/MyD88/NF‐κB/NLRP3 pathway.

## 4 DISCUSSION

Accumulating evidence have suggested the protective functions of emodin against lung injury. For example, emodin administration attenuates leukocyte infiltration and local hemorrhage and decreases the lung injury index in rats.^31^ Additionally, emodin administration increases the survival rate of the mice transfected with influenza A virus (IAV), attenuates pulmonary edema, and improves IAV‐induced histopathological changes.^32^ Moreover, emodin treatment improves the blood gas indexes and attenuates the lung edema in animals with pancreatitis‐stimulated lung injury.^33^ The findings of this study were consistent with the previous. Our study demonstrated that emodin administration increased the arterial oxygenation and dynamic compliance, decreased the airway pressures, and attenuated the alveolar damage and lung edema, indicating that emodin administration ameliorated lung injury caused by I/R.

LIRI is a complex, sterile inflammatory condition involving rapid oxidative stress and subsequent responses by all cells globally within the lung, ultimately leading to breakdown of the endothelial and epithelial barriers resulting in life‐threatening edema.^34^ The anti‐inflammatory efficacy of emodin has been reported in various diseases. For example, in mice with acute respiratory distress syndrome, emodin inhibits inflammatory response by inactivating NF‐κB.^35^ Additionally, emodin attenuates inflammation to alleviate the silica‐stimulated lung injury.^36^ Moreover, the anti‐inflammatory property of emodin is involved in preventing the pulmonary fibrosis.^37^ In the current study, we found the consistent results that emodin decreased the levels of IL‐6 and TNF‐α in lung tissues and serum of experimental I/R model and reduced the level of IL‐1β in OGD/R‐treated PMVECs.

Pyroptosis, which exhibits morphological characteristics of apoptosis and inflammatory necrosis, plays an important role in lung injury. LIRI could be alleviated by inhibiting the selective NLRP3 inflammasome, which is involved in the classical pyroptosis pathway.^14^ As reported, emodin inhibits pyroptosis in diverse pathological events. For example, in cardiomyocytes, emodin relieves myocardial I/R through inhibition of GSDMD‐mediated pyroptosis.^38^ Additionally, emodin attenuates inflammation and pyroptosis by inactivating methyltransferase‐like 3‐mediated NLRP3 in a cell model of sepsis brain injury.^39^ Moreover, emodin inhibits NLRP3 inflammasome‐mediated pyroptosis to alleviate lung injury.^40^ Camille et al. suggest the context‐dependent role of GSDMD in the release of IL‐1β.^41^ The current study revealed that LIRI in vivo and OGD/R in vitro both elevated levels of GSDMD‐N, caspase‐1 p20 and IL‐1β, which were decreased following emodin administration, suggesting that emodin attenuated the endothelial pyroptosis.

TLRs recruit MyD88, resulting in the activation of NF‐κB.^42^ Among these TLRs, TLR4 is the most important Toll homolog.^43^ Previous reports have suggested the interaction between emodin and TLR4/NF‐κB pathway. For example, emodin alleviates the intestinal and lung injury by inhibiting TLR4/NF‐κB pathway.^17^ Additionally, emodin inhibits influenza viral pneumonia by inactivating the TLR4/NF‐κB/MAPK pathways and activating Nrf2 signaling.^32^ Moreover, emodin attenuates the renal injury by downregulation of TLR4, MyD88 and NF‐κB levels.^44^ This study demonstrated that emodin reduced protein levels of TLR4, MyD88, and phosphorylated p65, suggesting that emodin inactivated the TLR4/NF‐κB pathway. Moreover, 5 μM of Bay‐117082 (NF‐κB inhibitor) reduced protein levels of caspase‐1 p20 and GSDMD‐N, indicating that NF‐κB inhibition attenuated the OGD/R‐induced pyroptosis of PMVECs.

A previous study has suggested that NLRP3 inhibition attenuates LIRI.^14^ The relationship between emodin and NLRP3 has been described previously. For example, emodin attenuates myocardial injury by inhibiting NLRP3 inflammasome activation.^28^ Additionally, emodin ameliorates the lung injury associated with acute pancreatitis by inactivating the NLRP3/IL‐1/CXCL1 signaling.^45^ Moreover, emodin protects hepatocytes from liver injury through inactivation of NLRP3 inflammasome, upregulation of Nrf2, and downregulation of the cGAS‐STING.^46^ In the current study, we found that emodin decreased the protein levels of NLRP3 and ASC in OGD/R‐treated PMVECs.

In summary, this study demonstrates that emodin protects against I/R‐induced lung injuries by inhibiting inflammation and endothelial pyroptosis in experimental I/R models. Mechanistically, the protective effects of emodin were associated with inactivation of the TLR4/MyD88/NF‐κB/NLRP3 pathway. There are also some limitations in this study. First, other animal models should be established before applying these results to human. Second, long‐term effects of different concentrations of emodin need further investigation. Nevertheless, we believed that emodin would be a promising agent for LIRI treatment.

## CONFLICT OF INTEREST

All authors have no conflict of interest to declare.

## ETHICS STATEMENT

Experimental protocols were granted approval from the Ethics Committee of Wuhan Myhalic Biotechnology Co., Ltd (approval number: 202104099).

## AUTHOR CONTRIBUTIONS

Tao Jin and Fen Ai conceived and designed the experiments. Tao Jin, Fen Ai, Jin Zhou, Lin Kong, Zhangming Xiong, Dingping Wang, Ruilin Lu, Zhen Chen, and Muxi Zhang carried out the experiments. Tao Jin, Fen Ai, and Muxi Zhang analyzed the data. Tao Jin, Fen Ai, and Muxi Zhang drafted the manuscript. All authors agreed to be accountable for all aspects of the work. All authors read and approved the final version of the manuscript.

## Data Availability

The data that support the findings of this study are available from the corresponding author upon reasonable request.

## References

[1] Pinto A , Immohr MB , Jahn A , et al. The extracellular isoform of superoxide dismutase has a significant impact on cardiovascular ischaemia and reperfusion injury during cardiopulmonary bypass. Eur J Cardiothorac Surg. 2016;50(6):1035‐1044. doi:10.1093/ejcts/ezw216 27999072

[2] Gielis JF , Beckers PAJ , Briedé JJ , Cos P , van Schil PE . Oxidative and nitrosative stress during pulmonary ischemia‐reperfusion injury: from the lab to the OR. Ann Transl Med. 2017;5(6):131. doi:10.21037/atm.2017.03.32 28462211 PMC5395487

[3] Leung CH , Caldarone CA , Wang F , et al. Remote ischemic conditioning prevents lung and liver injury after hemorrhagic shock/resuscitation: potential role of a humoral plasma factor. Ann Surg. 2015;261(6):1215‐1225. doi:10.1097/SLA.0000000000000877 25185480

[4] Deng C , Zhai Z , Wu D , et al. Inflammatory response and pneumocyte apoptosis during lung ischemia‐reperfusion injury in an experimental pulmonary thromboembolism model. J Thromb Thrombolysis. 2015;40(1):42‐53. doi:10.1007/s11239-015-1182-x 25677043 PMC4445764

[5] Lohser J , Slinger P . Lung injury after one‐lung ventilation: a review of the pathophysiologic mechanisms affecting the ventilated and the collapsed lung. Anesth Analg. 2015;121(2):302‐318. doi:10.1213/ANE.0000000000000808 26197368

[6] Nakazawa D , Kumar SV , Marschner J , et al. Histones and neutrophil extracellular traps enhance tubular necrosis and remote organ injury in ischemic AKI. J am Soc Nephrol. 2017;28(6):1753‐1768. doi:10.1681/ASN.2016080925 28073931 PMC5461800

[7] Grootjans S , Vanden Berghe T , Vandenabeele P . Initiation and execution mechanisms of necroptosis: an overview. Cell Death Differ. 2017;24(7):1184‐1195. doi:10.1038/cdd.2017.65 28498367 PMC5520172

[8] Bergsbaken T , Fink SL , Cookson BT . Pyroptosis: host cell death and inflammation. Nat Rev Microbiol. 2009;7(2):99‐109. doi:10.1038/nrmicro2070 19148178 PMC2910423

[9] Ding J , Wang K , Liu W , et al. Pore‐forming activity and structural autoinhibition of the gasdermin family. Nature. 2016;535(7610):111‐116. doi:10.1038/nature18590 27281216

[10] Kovacs SB , Miao EA . Gasdermins: effectors of pyroptosis. Trends Cell Biol. 2017;27(9):673‐684. doi:10.1016/j.tcb.2017.05.005 28619472 PMC5565696

[11] He Y , Zeng MY , Yang D , Motro B , Núñez G . NEK7 is an essential mediator of NLRP3 activation downstream of potassium efflux. Nature. 2016;530(7590):354‐357. doi:10.1038/nature16959 26814970 PMC4810788

[12] Yuan J , Najafov A , Py BF . Roles of caspases in necrotic cell death. Cell. 2016;167(7):1693‐1704. doi:10.1016/j.cell.2016.11.047 27984721 PMC5381727

[13] Shi J , Zhao Y , Wang K , et al. Cleavage of GSDMD by inflammatory caspases determines pyroptotic cell death. Nature. 2015;526(7575):660‐665. doi:10.1038/nature15514 26375003

[14] Xu KY , Wu CY , Tong S , Xiong P , Wang SH . The selective Nlrp3 inflammasome inhibitor Mcc950 attenuates lung ischemia‐reperfusion injury. Biochem Biophys Res Commun. 2018;503(4):3031‐3037. doi:10.1016/j.bbrc.2018.08.089 30146255

[15] Wu S , Li Z , Ye M , et al. VX765, a specific caspase‐1 inhibitor, alleviates lung ischemia reperfusion injury by suppressing endothelial pyroptosis and barrier dysfunction. Biomed Res Int. 2021;2021:4525988. 34977239 10.1155/2021/4525988 PMC8716216

[16] Wu Z , Chen Q , Ke D , Li G , Deng W . Emodin protects against diabetic cardiomyopathy by regulating the AKT/GSK‐3β signaling pathway in the rat model. Molecules. 2014;19(9):14782‐14793. doi:10.3390/molecules190914782 25232702 PMC6271268

[17] Qian J , Li G , Jin X , et al. Emodin protects against intestinal and lung injury induced by acute intestinal injury by modulating SP‐A and TLR4/NF‐κB pathway. Biosci Rep. 2020;40(9):BSR20201605. doi:10.1042/BSR20201605 32915230 PMC7517261

[18] Gao Z , Sui J , Fan R , Qu W , Dong X , Sun D . Emodin protects against acute pancreatitis‐associated lung injury by inhibiting NLPR3 inflammasome activation via Nrf2/HO‐1 signaling. Drug des Devel Ther. 2020;14:1971‐1982. doi:10.2147/DDDT.S247103 PMC7247729 32546964

[19] Li X , Shan C , Wu Z , Yu H , Yang A , Tan B . Emodin alleviated pulmonary inflammation in rats with LPS‐induced acute lung injury through inhibiting the mTOR/HIF‐1α/VEGF signaling pathway. Inflamm Res. 2020;69(4):365‐373. doi:10.1007/s00011-020-01331-3 32130427

[20] Dong Y , Zhang L , Jiang Y , Dai J , Tang L , Liu G . Emodin reactivated autophagy and alleviated inflammatory lung injury in mice with lethal endotoxemia. Exp Anim. 2019;68(4):559‐568. doi:10.1538/expanim.19-0004 31292306 PMC6842802

[21] Wang Y , Liu Q , Cai J , et al. Emodin prevents renal ischemia‐reperfusion injury via suppression of CAMKII/DRP1‐mediated mitochondrial fission. Eur J Pharmacol. 2022;916:174603. doi:10.1016/j.ejphar.2021.174603 34793771

[22] Du Y , Ko KM . Effects of pharmacological preconditioning by emodin/oleanolic acid treatment and/or ischemic preconditioning on mitochondrial antioxidant components as well as the susceptibility to ischemia‐reperfusion injury in rat hearts. Mol Cell Biochem. 2006;288(1–2):135‐142. doi:10.1007/s11010-006-9129-3 16583138

[23] Leung SW , Lai JH , Wu JCC , et al. Neuroprotective effects of emodin against ischemia/reperfusion injury through activating ERK‐1/2 signaling pathway. Int J Mol Sci. 2020;21(8):2899. doi:10.3390/ijms21082899 32326191 PMC7215870

[24] Bauernfeind FG , Horvath G , Stutz A , et al. Cutting edge: NF‐κB activating pattern recognition and cytokine receptors license NLRP3 inflammasome activation by regulating NLRP3 expression. J Immunol. 2009;183(2):787‐791. doi:10.4049/jimmunol.0901363 19570822 PMC2824855

[25] Akira S , Uematsu S , Takeuchi O . Pathogen recognition and innate immunity. Cell. 2006;124(4):783‐801. doi:10.1016/j.cell.2006.02.015 16497588

[26] Ve T , Gay NJ , Mansell A , Kobe B , Kellie S . Adaptors in toll‐like receptor signaling and their potential as therapeutic targets. Curr Drug Targets. 2012;13(11):1360‐1374. doi:10.2174/138945012803530260 22664090

[27] Ve T , Vajjhala PR , Hedger A , et al. Structural basis of TIR‐domain‐assembly formation in MAL‐ and MyD88‐dependent TLR4 signaling. Nat Struct Mol Biol. 2017;24(9):743‐751. doi:10.1038/nsmb.3444 PMC8059215 28759049

[28] Dai S , Ye B , Chen L , Hong G , Zhao G , Lu Z . Emodin alleviates LPS‐induced myocardial injury through inhibition of NLRP3 inflammasome activation. Phytother Res. 2021;35(9):5203‐5213. doi:10.1002/ptr.7191 34131970

[29] Wu SY , Tang SE , Ko FC , Wu GC , Huang KL , Chu SJ . Valproic acid attenuates acute lung injury induced by ischemia‐reperfusion in rats. Anesthesiology. 2015;122(6):1327‐1337. doi:10.1097/ALN.0000000000000618 25749053

[30] Wang T , Liu C , Pan LH , et al. Inhibition of p38 MAPK mitigates lung ischemia reperfusion injury by reducing blood‐air barrier hyperpermeability. Front Pharmacol. 2020;11:569251. doi:10.3389/fphar.2020.569251 PMC7759682 33362540

[31] Guo R , Li Y , Han M , Liu J , Sun Y . Emodin attenuates acute lung injury in cecal‐ligation and puncture rats. Int Immunopharmacol. 2020;85:106626. doi:10.1016/j.intimp.2020.106626 32492627

[32] Dai JP , Wang QW , Su Y , et al. Emodin inhibition of influenza A virus replication and influenza viral pneumonia via the Nrf2, TLR4, p38/JNK and NF‐κB pathways. Molecules. 2017;22(10):1754. doi:10.3390/molecules22101754 PMC6151665 29057806

[33] Xu J , Huang B , Wang Y , et al. Emodin ameliorates acute lung injury induced by severe acute pancreatitis through the up‐regulated expressions of AQP1 and AQP5 in lung. Clin Exp Pharmacol Physiol. 2016;43(11):1071‐1079. doi:10.1111/1440-1681.12627 27452155

[34] Laubach VE , Sharma AK . Mechanisms of lung ischemia‐reperfusion injury. Curr Opin Organ Transplant. 2016;21(3):246‐252. doi:10.1097/MOT.0000000000000304 PMC4861054 26945320

[35] Liu B , Cheng Y , Wu Y , et al. Emodin improves alveolar hypercoagulation and inhibits pulmonary inflammation in LPS‐provoked ARDS in mice via NF‐κB inactivation. Int Immunopharmacol. 2020;88:107020. doi:10.1016/j.intimp.2020.107020 33182048

[36] Pang X , Shao L , Nie X , et al. Emodin attenuates silica‐induced lung injury by inhibition of inflammation, apoptosis and epithelial‐mesenchymal transition. Int Immunopharmacol. 2021;91:107277. doi:10.1016/j.intimp.2020.107277 33352442

[37] Tian SL , Yang Y , Liu XL , Xu QB . Emodin attenuates bleomycin‐induced pulmonary fibrosis via anti‐inflammatory and anti‐oxidative activities in rats. Med Sci Monit. 2018;24:1‐10. doi:10.12659/MSM.905496 PMC5759514 29290631

[38] Ye B , Chen X , Dai S , et al. Emodin alleviates myocardial ischemia/reperfusion injury by inhibiting gasdermin D‐mediated pyroptosis in cardiomyocytes. Drug des Devel Ther. 2019;13:975‐990. doi:10.2147/DDDT.S195412 PMC6438141 30988600

[39] Wang B , Liu Y , Jiang R , et al. Emodin relieves the inflammation and pyroptosis of lipopolysaccharide‐treated 1321N1 cells by regulating methyltransferase‐like 3 ‐mediated NLR family pyrin domain containing 3 expression. Bioengineered. 2022;13(3):6740‐6749. doi:10.1080/21655979.2022.2045836 PMC8973593 35246004

[40] Liu Y , Shang L , Zhou J , Pan G , Zhou F , Yang S . Emodin attenuates LPS‐induced acute lung injury by inhibiting NLRP3 inflammasome‐dependent pyroptosis signaling pathway in vitro and in vivo. Inflammation. 2021;45:753‐767. 10.1007/s10753-021-01581-1 PMC8956541 34787801

[41] Chauvin C , Retnakumar SV , Bayry J . Gasdermin D as a cellular switch to orientate immune responses via IL‐33 or IL‐1β. Cell Mol Immunol. 2022;20(1):1‐3. doi:10.1038/s41423-022-00950-6 PMC9664042 36380096

[42] Chu Q , Sun Y , Cui J , Xu T . MicroRNA‐3570 modulates the NF‐κB pathway in teleost fish by targeting MyD88. J Immunol. 2017;198(8):3274‐3282. doi:10.4049/jimmunol.1602064 28250156

[43] Zhu Y , Tong Q , Ye J , et al. Nogo‐B facilitates LPS‐mediated immune responses by up‐regulation of TLR4‐signaling in macrophage RAW264.7. Cell Physiol Biochem. 2017;41(1):274‐285. doi:10.1159/000456094 28214833

[44] Lu Z , Ji C , Luo X , et al. Nanoparticle‐mediated delivery of emodin via colonic irrigation attenuates renal injury in 5/6 nephrectomized rats. Front Pharmacol. 2020;11:606227. 10.3389/fphar.2020.606227 PMC7858270 33551808

[45] Xu Q , Wang M , Guo H , et al. Emodin alleviates severe acute pancreatitis‐associated acute lung injury by inhibiting the cold‐inducible RNA‐binding protein (CIRP)‐mediated activation of the NLRP3/IL‐1β/CXCL1 signaling. Front Pharmacol. 2021;12:655372. doi:10.3389/fphar.2021.655372 PMC8103163 33967799

[46] Shen P , Han L , Chen G , Cheng Z , Liu Q . Emodin attenuates acetaminophen‐induced hepatotoxicity via the cGAS‐STING pathway. Inflammation. 2022;45(1):74‐87. doi:10.1007/s10753-021-01529-5 34409550

